# Immunity to RSV in Early-Life

**DOI:** 10.3389/fimmu.2014.00466

**Published:** 2014-09-29

**Authors:** Laura Lambert, Agnes M. Sagfors, Peter J. M. Openshaw, Fiona J. Culley

**Affiliations:** ^1^National Heart and Lung Institute, Imperial College London, London, UK

**Keywords:** respiratory, neonatal, RSV, mucosal immunology, bronchiolitis, viral

## Abstract

Respiratory Syncytial Virus (RSV) is the commonest cause of severe respiratory infection in infants, leading to over 3 million hospitalizations and around 66,000 deaths worldwide each year. RSV bronchiolitis predominantly strikes apparently healthy infants, with age as the principal risk factor for severe disease. The differences in the immune response to RSV in the very young are likely to be key to determining the clinical outcome of this common infection. Remarkable age-related differences in innate cytokine responses follow recognition of RSV by numerous pattern recognition receptors, and the importance of this early response is supported by polymorphisms in many early innate genes, which associate with bronchiolitis. In the absence of strong, Th1 polarizing signals, infants develop T cell responses that can be biased away from protective Th1 and cytotoxic T cell immunity toward dysregulated, Th2 and Th17 polarization. This may contribute not only to the initial inflammation in bronchiolitis, but also to the long-term increased risk of developing wheeze and asthma later in life. An early-life vaccine for RSV will need to overcome the difficulties of generating a protective response in infants, and the proven risks associated with generating an inappropriate response. Infantile T follicular helper and B cell responses are immature, but maternal antibodies can afford some protection. Thus, maternal vaccination is a promising alternative approach. However, even in adults adaptive immunity following natural infection is poorly protective, allowing re-infection even with the same strain of RSV. This gives us few clues as to how effective vaccination could be achieved. Challenges remain in understanding how respiratory immunity matures with age, and the external factors influencing its development. Determining why some infants develop bronchiolitis should lead to new therapies to lessen the clinical impact of RSV and aid the rational design of protective vaccines.

## Introduction

Respiratory syncytial virus (RSV) was first isolated in 1956 from a captive chimpanzee (1). Initially named Chimpanzee Coryza Agent, RSV was soon recovered from infants with lower respiratory illness and identified as a human pathogen (2). Almost six decades later, it remains the commonest viral cause of serious respiratory illness in children under 5 years of age and the major cause of infantile bronchiolitis. RSV is estimated to cause 3.4 million hospitalizations and at least 66,000 deaths worldwide each year (3, 4), but since 99% of these deaths occur in developing countries, this is likely to be an underestimate. Immunity to reinfection with a single strain of RSV is, at best, partial; re-infections with antigenically similar strains occur throughout life and through to old age. The continued lack of any specific treatment or a safe and effective vaccine indicates the vital importance of better understanding of immunity to RSV infection in making progress in reducing global mortality and morbidity from RSV infection.

## Virus

**Figure 1:**
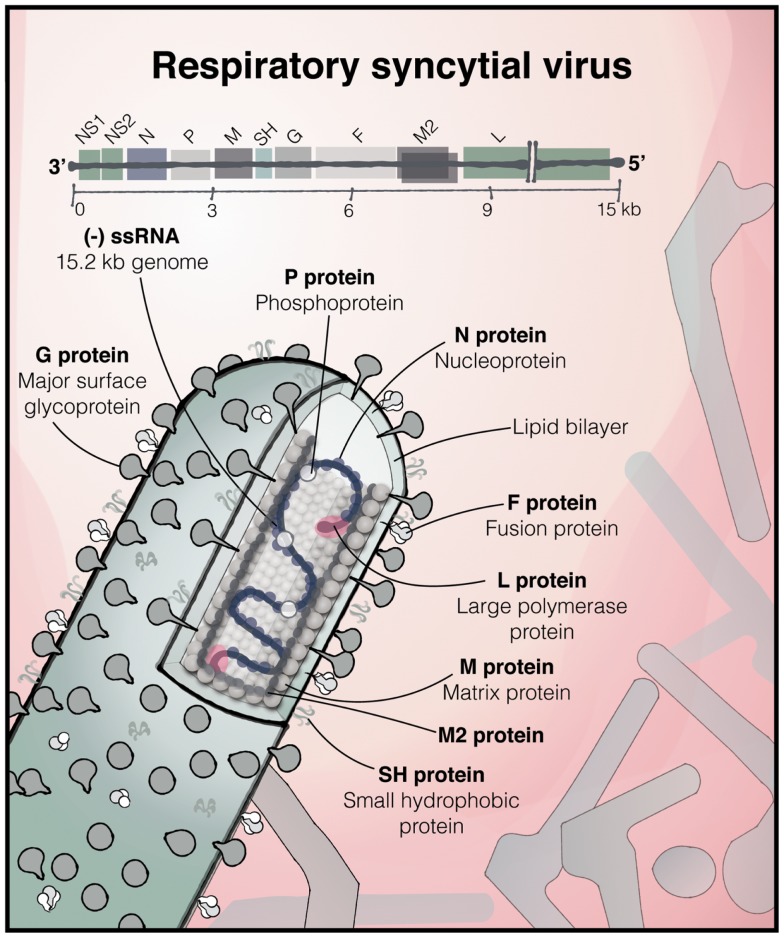
**The structure of RSV**. The 15.2 kb negative sense, single stranded RNA RSV genome consisting of 10 genes, encoding 11 proteins, and below, an illustration of a filamentous virus particle; one of the predominant forms, which bud from the infected cell. The outer envelope contains the heavily glycosylated surface glycoprotein G and the fusion (F) and SH proteins. The matrix protein lies within the membrane, surrounding the ribonucleoprotein complex, consisting of the genome associated with N, P, and the large RNA-dependent RNA polymerase (L) protein [based on (6) and (7)].

Respiratory syncytial virus, of the family *Paramyxoviridae*, order *Mononegavirales*, is an enveloped, non-segmented negative-strand RNA virus (Figure 1). One of the more complex members of the *Paramyxoviridae* family, its 15.2 kb genome comprises 10 genes in the order 3′-NS1-NS2-N-P-M-SH-G-F-M2-L-5′. These encode a total of 11 proteins, as the M2 mRNA contains two overlapping open reading frames resulting in two polypeptides, M2-1 and M2-2. The two major surface proteins of RSV, the F and highly glycosylated G-protein are believed to be the major targets of the antibody response. Antisera to RSV show extensive cross-reactivity to natural strains, but two major antigenic subgroups have been defined [A and B; (5)]. The relative antigenic stability of RSV makes the apparent lack of effective immunological memory all the more intriguing. Infection is normally confined to the respiratory mucosa and does not usually disseminate to other organs or appear in the blood.

## Clinical Disease and Treatment

By the age of two, over 80% of children have experienced at least one RSV infection, 2/3 of these occurring in the first year of life (8). Whilst the majority of infants display only mild upper respiratory tract infection (URTI) or occasionally otitis media, around one-third will develop an infection of the lower respiratory tract (LRTI), usually bronchiolitis. This is caused by an infiltration of inflammatory cells into the airspaces, mucus hyper-production, shedding of necrotic airway epithelial cells, and edema of the airway wall. These processes lead to a narrowing of the airway lumen, airflow obstruction, overinflation, and impaired gas exchange. In more severe RSV disease crackles and wheeze occur with labored breathing, tachypnea, and hypoxia (9). In children under 5 years of age, around 10% of those with RSV LRTI require hospitalization (3). The peak of admissions in the UK occurs at approximately 1 month of age (10). In addition to the enormous pediatric burden, RSV is increasingly recognized as an important pathogen of the elderly, causing a mortality rate approaching that of influenza A in the over-65s (11, 12).

Palivizumab (Synagis) is a humanized monoclonal antibody against the F protein of RSV. It is given prophylactically to infants at high risk and protects against severe disease (13), but has no benefit in those with active infection. The anti-viral drug ribavirin is of limited efficacy (14).

### Risk factors

One of the key unanswered questions is why some unfortunate infants develop severe bronchiolitis, while most suffer mild URTI or LRTI. Many risk factors have been defined including prematurity, low birth weight, male sex, low socio-economic status, and pre-existing medical conditions such as congenital heart disease and immunodeficiency (4, 15). HIV infection is associated with increased risk of RSV LRTI and poor outcome, and in such children seasonal peaks of RSV infection are less evident. This may in part explain the large disease burden in areas of high HIV-prevalence (16, 17). Despite the known predictors of severe disease, the majority of infants hospitalized with RSV bronchiolitis are previously healthy and without known risk factors other than young age, the biggest risk factor for severe bronchiolitis (4, 10, 18, 19).

It is likely that maternal factors may influence the developing lungs and immune system of the fetus. Maternal smoking and diet are independent risk factors for the development of bronchiolitis (20, 21) and low cord blood vitamin D in otherwise healthy infants is associated with a sixfold increased risk of developing RSV LRTI in the first year of life (22) consistent with a linkage of bronchiolitis with polymorphisms in the vitamin D receptor (23). *In utero* influences on infant immune responses and the outcome of RSV infection is an area that deserves further study.

**Figure 2:**
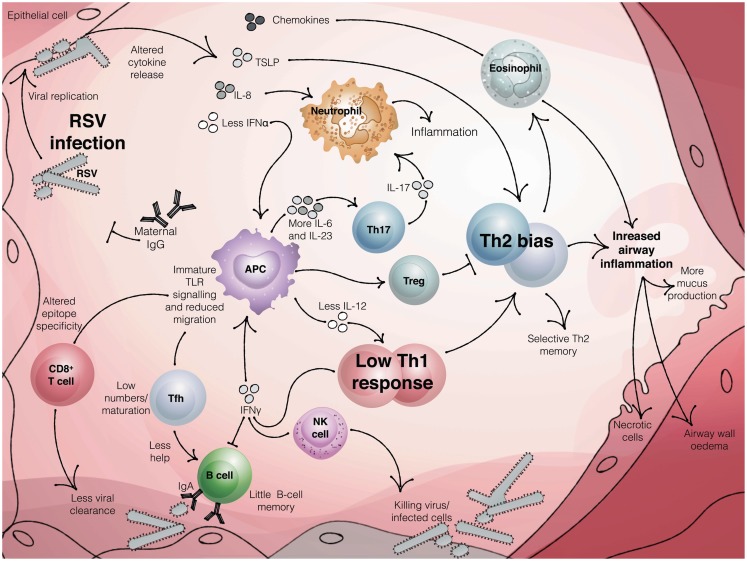
**The neonatal immune response to RSV infection**. Maternal antibody can reduce the burden of RSV infection in infants. Once infection is established, the innate immune response produces reduced levels of antiviral cytokines, such as interferons. In infants, reduced signaling from TLRs and altered antigen presenting cell function, including low IL-12 and enhanced production of IL-6 and IL-23, coupled with a reduced activation of regulatory T cells, may result in an adaptive response that is skewed toward Th2 and Th17 and away from protective Th1 and CTL. Impaired Tfh activation, coupled with little or no B cell memory and inhibition of antibody production by IFNγ, produces low titer, low affinity antibody. The result may be a poorly protective and dysregulated immune response that leads to bronchiolitis in susceptible infants.

Around 20% of the susceptibility to severe RSV has been attributed to genetic factors (24) and numerous genetic polymorphisms in immune genes have been reported. These include variants in the 5q31 Th2 cytokine locus (25), IL-8 (26), and IL-4-Rα (27, 28). A large cohort study found that single nucleotide polymorphisms in genes of the innate immune system were particularly associated with hospitalization with bronchiolitis (23). A small number of genes were found to be differentially expressed in cord blood from infants who went on to be hospitalized with RSV versus those that experienced a mild infection, including *TNFRSF25*, which is involved in activation of NF-κB (29). Consistent with this, *NFKBIA* promoter variants which increase toll-like receptor (TLR)-mediated inflammatory cytokine production are associated with severe RSV bronchiolitis and AHR in children with RSV in the first year of life (30). Together this suggests that the immune response to RSV is key to determining the outcome of infection in the very young (Figure 2).

## Immune Response to RSV Infection

It is difficult to overcome the ethical and practical problems associated with studying immune responses in neonates, especially in a transient self-limiting disease from which full recovery is the rule. We therefore have relatively little knowledge of what the normal infantile immune response to RSV comprises, especially at the site of disease rather than in peripheral blood. Instead, much of the information available comes from animal studies, clinical studies in adults, or very severe or fatal cases of infection in babies, often with the complications of comorbidities such as bacterial superinfection. Histopathological analysis suggests that the virus predominantly infects the superficial ciliated cells of the upper airway, the epithelium of the small bronchioles and type-I alveolar pneumocytes (31). The fact that the virus completes it life cycle in superficial airway cells may aid its immune evasion, since systemic immunity normally gains little access to the epithelium.

The exact contributions of virus and host to the pathogenesis of bronchiolitis remain contentious. Histological studies show a pronounced inflammatory infiltrate in bronchiolitis and animal models support a role for immune mediated pathology to RSV (31–33). However, others report that severe disease is associated with a high viral load and relative lack of lymphocytes in the lungs (34).

## Detection of the Virus and the Early Innate Response

Adaptive immune responses have been the focus of the ongoing quest for vaccines. However, most of the SNPs linked to infant RSV bronchiolitis identify the critical importance of the innate immune system (23), indicating that this early response to the virus could have important consequences for the outcome of the infection.

Early viral detection is mediated through pattern recognition receptors (PRRs) expressed by epithelial cells, fibroblasts, and antigen presenting cells (APCs) of the respiratory tract. Their ligation results in activation of NF-κB and the interferon regulatory factor (IRF) family members, leading to upregulated expression of cytokines, chemokines, and anti-viral factors. PRRs known to be important for recognition of RSV are TLR-4, TLR3, TLR2/6, TLR7/8, and RIG-I [reviewed (35, 36)].

The first PRR to be implicated in the response to RSV was TLR-4. Kurt-Jones and colleagues demonstrated *in vitro* that the F protein could trigger IL-6 production from human PBMCs, dependent on TLR-4/CD14 engagement (37). The same group showed impaired viral clearance and natural killer (NK) cell responses in a *Tlr4* knock-out mouse, suggesting a protective *in vivo* role of TLR-4 in RSV infection (38). In humans, there are two common missense mutations in the *Tlr4* gene, associated with hyporesponsiveness to intranasal LPS (39, 40). Both of these polymorphisms have subsequently been linked to increased risk of severe RSV bronchiolitis in infants (41), and *in vitro* cytokine production was blunted in PMBCs and human bronchial epithelial cells exposed to RSV if they expressed these TLR-4 alleles (42). This genetic association with severe RSV disease was not confirmed by Paulus et al. (43), and another study found associations only when their cohort was divided into cases from two different RSV epidemics (44). Thus, it may be that particular RSV strains and TLR-4 phenotypes can combine to influence risk of severe bronchiolitis.

Initial detection of RSV is also mediated by TLR3, which is expressed intracellularly and recognizes dsRNA present during RSV replication. In contrast to the *Tlr4* knock-out model, *Tlr3* knock-out mice can still clear RSV effectively; however, they display goblet cell hyperplasia with mucus over-production (45), which in the infantile lung could result in a more severe disease phenotype given the narrow lumens of the immature respiratory tract. Knocking out TLR7, which along with TLR8 detects ssRNA, also resulted in exacerbated mucus production in a mouse model (46). Interestingly, the lack of TLR7 seemed to skew the RSV T cell response toward Th17, which is associated with immunopathology in human infants (see below).

Given the exacerbated pathology seen in animal models lacking particular PRRs, it is tempting to relate these findings to immaturities in human neonatal PRR responses. In stimulation assays of cord blood versus adult blood cells using TLR ligands, Kollmann et al. reported qualitative and quantitative differences in the responses of adults and neonates, with neonatal monocytes producing more Th17-skewing cytokines (47). Another recent study found neonatal blood contained a lower proportion of TLR-4^hi^ CD14^+^ CD16^+^ monocytes, leading to lower overall blood TLR-4 expression (48). Since TLR responses appear to be important for determining the outcome of an RSV infection, these age-dependent differences could help to explain the striking susceptibility of the very young to severe RSV disease.

Although these studies of cord blood are informative, the innate immune system of the lung is likely to be different in many critical aspects to that of the systemic immune system. We currently know remarkably little about how innate pulmonary immunity matures with age and the factors influencing it. This is partly because of the difficulty of accessing structural lung cells in children with bronchiolitis and healthy controls. Extensive epithelial sloughing is associated with severe RSV disease and as the primary site of replication of RSV the respiratory epithelium is thought to be an important early source of cytokines. We are just beginning to understand how the respiratory epithelium differs in infants and how it responds to viral infection (49). Recent studies of well differentiated primary pediatric nasal and bronchial epithelial cells have demonstrated viral growth and shedding in these cells and found that the extent of epithelial production of cytokines and mucus and epithelial apoptosis differs with viral strains (50–53). Importantly, responses in nasally derived epithelial cells were similar to those from the lower airways, suggesting that these may be a more easily accessible source of respiratory epithelial cells with which to study responses to RSV in infants.

Once the immune system detects the presence of virus, production of pro-inflammatory cytokines and chemokines is initiated. This leads to a positive feedback loop whereby cells recruited into the airways contribute to mediator production. For example, high concentrations of the pro-inflammatory cytokines IL-6 and TNF-α are present in the bronchoalveolar lavage (BAL) fluid from infants with RSV bronchiolitis (54) and the production of chemokines such as CXCL10 (IP-10), CXCL8 (IL-8), CCL2 (MCP1), CCL3 (MIP-1α), and CCL5 (RANTES) has also been demonstrated (55).

In the early stages of bronchiolitis, it is thought that neutrophils are the major recruited leukocyte to both the upper and lower airway (56, 57). Whether this significant neutrophilia is pathogenic or protective is debated – whilst neutrophils can destroy infected cells, they can also release enzymes that damage surrounding tissue. A genetic polymorphism in the IL-8 promoter region, linked to augmented IL-8 production on whole-blood LPS stimulation, has been associated with increased risk of severe RSV disease in infants, implicating this potent neutrophil chemoattractant in pathogenesis (26). Increased levels of IL-8 have been detected in the nasal lavage of infants hospitalized with RSV-positive compared to RSV-negative respiratory illnesses (58). In a recent study, Mella et al. also found higher levels of IL-8 and IL-6 in the serum of infants hospitalized with RSV compared to healthy controls; however, when blood was stimulated *in vitro*, the production of these cytokines negatively correlated with disease severity (59). It is not clear whether neutrophilia is also seen in common milder cases of bronchiolitis.

In the early response to viral infection, another key set of inflammatory mediators to be induced is the interferon (IFN) family. Type-I IFNs (IFN-α and IFN-β) act on the IFNAR receptor, found on all cell types, to induce an anti-viral state in the cell. One way in which RSV may subvert effective viral clearance is via its NS1 and 2 proteins, which inhibit IFN-α/β signaling, suggesting that a more powerful innate IFN response would be detrimental to viral survival (60–65). Indeed, type-I IFN signaling profoundly influences the outcome of RSV infection in animal models, and mice lacking IFNAR show increased viral load but decreased production of pro-inflammatory cytokines, implying that type-I IFNs are important players in initiation of the innate response (66, 67). Plasmacytoid dendritic cells (pDCs), are potent producers of IFN-α/β. These cells are mobilized, along with myeloid DCs, to the nasal mucosa during RSV infection (68) and according to murine studies are important for both clearance of the virus and avoidance of excessive immunopathology (69, 70). However, in neonatal cord blood, pDCs have been shown to be deficient in their ability to produce IFN-α in response to RSV, which could potentiate the risk of LRTI in early-life (71).

Mouse models have allowed study of lung-resident DCs and the identification of immaturities of lung dendritic cells in the neonatal response to RSV. Ruckwardt and colleagues reported an imbalance in the subsets of two lung-resident DCs in neonatally infected mice, with an increased ratio of CD103^+^:CD11c^+^ cells found in the draining lymph nodes. These neonatal DCs also expressed lower levels of co-stimulatory molecules and were less able to process and transport antigen (72). Coupled with an ability of the RSV NS1/2 proteins to inhibit DC maturation (probably via the IFN antagonism already described) (73), RSV appears particularly well-equipped in an already immature host to avoid effective antigen presentation and to alter subsequent T cell priming.

Natural killer cells are also an important part of the early immune response according to the murine model, providing an early source of IFN-γ to help activate DCs and prime the T cell response, and via direct cytotoxic destruction of infected cells (74, 75). Again, the contribution of these cells to protection versus immunopathology is debateable. In infants, severe RSV bronchiolitis has been associated with low NK cell numbers in the lung and the peripheral blood, suggesting a protective effect (34, 76). Low levels of NK cells might also be due to the fact that G-protein of RSV is able to compete with fractalkine, one of the chemokines that attracts these cells, for its receptor (CX3CR1) (77). From studies on cord blood, neonatal NK cells are thought to be immature, expressing lower levels of certain inhibitory and activating receptors and of IFN-γ (78). In animal models, NK cells in the BAL peak at day 3 post-RSV infection (79), and appear to be vital for development of an “appropriate” T cell response (discussed below) and the avoidance of Th2-skewing and allergic sensitization (74).

## Adaptive Immunity to RSV and Vaccination

Natural infection gives us few clues about adaptive protection against RSV. Following infection, protection generated is short-lived and incomplete, allowing RSV to re-infect the host throughout life (80). Indeed, it is possible, experimentally, to repeatedly re-infect an individual with an identical RSV strain (81). Most natural re-infections occur with genetically different strains, although whether this is due to the host immune response and infection history, or whether this simply reflects changes in circulating virus, is unclear. Recent longitudinal studies of RSV infections in children over a number of RSV seasons established that children can be naturally re-infected with the same strain of virus even within the same cold season (82), although the rate of a second RSV infection is reduced for 6 months (83) and the duration of viral shedding is reduced (84), suggesting some generation of partially protective immunity in infants following natural infection. The fact that dominant circulating strains of RSV are replaced in successive epidemics suggests the existence of some strain-specific immunity at a population level that is sufficient to confer a competitive advantage on a new strain. An outstanding question is whether infants develop more severe disease because of the immaturity of their immune system or because they are undergoing a primary infection. Severe disease is evident in children undergoing a secondary RSV infection at a young age (82, 83) suggesting that immunity generated after natural infection does not confer protection against severe disease and that age, rather than experience of infection, is the most important factor in disease severity.

### B cell response to RSV infection

The remarkable effectiveness of palivizumab prophylaxis shows that neutralizing antibody of sufficiently high affinity and titer is sufficient to confer protection of the lower airway. What is less clear is why antibody produced after natural infection is poorly protective against reinfection. In adults, the highest titers of naturally occurring pre-existing serum neutralizing IgG and nasal IgA correlate with protection against naturally acquired infection (85, 86), and with protection in re-challenge studies (81). Further, those with the highest levels of neutralizing antibody tend to be protected against RSV-associated LRTI and hospitalization (87), but in most adults antibody titers are below the levels needed to achieve full airway protection despite a lifetime of repeated infection. It is possible that a high antibody titer is simply indicative of recent infection and that it is a surrogate marker of some other response that is protective.

In humans, IgG antibody to RSV is passed from the mother to the infant *via* the placenta and colostrum and in the first few months of life the majority of antibody present in the neonatal serum will be maternal IgG. A study of a large birth cohort in Kenya demonstrated that 97% of infants had RSV-specific antibody from their mothers, but this declines rapidly with a half-life of 2–3 months (88, 89). The titer of maternal antibody correlates with protection against infection, severe disease, and hospitalization (83, 88, 90–92).

### Vaccination to generate protective antibody responses

Following a disastrous trial of formalin-inactivated-RSV (FI-RSV) vaccine in the 1960s, research into the development of an RSV vaccine has proceeded cautiously and no vaccine for RSV has yet been licensed (93–95). Children who had received the FI-RSV vaccine had a far higher incidence of severe disease, and two died, as a consequence of natural RSV infection following vaccination. The most significant incidence of enhanced disease occurred in the youngest infants, which may indicate that the immaturity of the infant immune system or the lack of previous experience of infection contributed to the vaccine enhanced illness. As these vaccine trials occurred over 50 years ago, we are not able to precisely elucidate what went wrong, but the mouse and primate models largely recapitulate the eosinophilic inflammation, which was reported in the post-mortem examination of the fatal cases. The antibodies induced in vaccinated children were poorly neutralizing and formation of immune complexes and local complement activation in the lungs may have promoted pathology (96, 97). Although they may not entirely recapitulate the clinical situation in the original trials (98), studies in mice have suggested reasons for the poor quality of the antibody response, including modification of viral proteins by the formalin treatment (99) and the lack of a strong TLR stimulus in FI-RSV (100). Further, they have shown the regulatory T cell response following FI-RSV vaccination and infection is defective (101). Thus properties of the FI-RSV preparation combined with the immature infant immune system led to devastating results.

Maternal vaccination to induce sufficiently high titer of antibody could be a safe and effective strategy to delay infection and protect against severe disease within the first few months of life. A small scale randomized, double-blind, placebo controlled study of healthy women immunized during the third trimester of pregnancy demonstrated that maternal vaccination could boost antibody titers of RSV in the mother and infants, with no apparent adverse effects (102).

Infants are vulnerable to RSV infection once maternal antibody has waned and so those 4 months old and above are important candidates for the development of an effective vaccine (4, 98, 103) and a number of promising vaccine candidates are being developed by pharmaceutical companies (104). Most research into the development of an RSV vaccine has focused on the generation of protective antibodies. In particular, research has centered on generating antibodies to the pre-fusion conformation of the F protein, the structural form present on the surface of the free virus particle, which contains unique antigenic sites recognized by neutralizing antibodies [(105) and (106)]. Neonates can generate their own antibody response following RSV infection, although this is low or absent in the youngest children (107–109). In autopsy samples of infants who had died of bronchiolitis, large numbers of infiltrating IgA, IgG, and IgM secreting B cells were found in the lungs (109). In bronchiolitis, the local nasal IgA and IgM response, but not IgG, and production of the B cell tropic factor APRIL, correlate with better oxygen saturation, suggesting a protective role for local secreted antibody (109). Interestingly, the repertoire of V-gene segments used by young infants <3 months of age to generate RSV-specific antibodies is biased compared to those used in adults, with fewer somatic mutations, and this may account for the poor functional quality of the antibody response at this age (110).

Any neonatal RSV vaccine will have to overcome the limitations of the neonatal antibody response to produce an antibody of sufficiently high quantity and quality to prevent severe disease. The development of effective vaccines for neonates is particularly challenging, for many reasons. Passively received antibodies from the mother, while providing protection against infection, can impair responses to vaccine antigens, perhaps by sequestering antigen and effectively reducing the antigenic load, or by masking the relevant protective epitopes from the B cell receptor, leading to the generation of antibody responses to sub-optimal epitopes (111, 112). This may be one reason for a low antibody production to RSV in neonates and there is some evidence of a reciprocal relationship between maternally derived serum IgG and the production of nasal sIgA in the neonate following primary infection (108).

The immaturity of the neonatal immune system means that even in the absence of any interference by maternal antibody, responses to vaccines are often weak, inappropriately polarized and short-lived (113, 114). Measles, like RSV, is a paramyxovirus spread through the respiratory tract, which can cause severe disease in infants, but for which there is an effective vaccine. Even in the absence of passively acquired antibodies, measles vaccination resulted in significantly lower neutralizing antibody titer in infants below 6 months of age compared to those 9 months old and above, with only 36% of infants of 6 months old achieving protective titers, compared to 100% of older infants (115, 116). This indicates that even once an effective vaccine is developed, the immaturity of the neonatal immune system itself is a barrier to generation of protective immunity.

A protective vaccine for neonates will need to overcome the intrinsic differences in neonatal B cells, as well as providing adequate and appropriate T cell help for the B cells to become activated (117). Studies of splenic B cells in mice and blood B cells in human neonates reveal reduced expression of co-stimulatory molecules, CD40, CD80, and CD86 and TACI (transmembrane activator and calcium-modulating cyclophilin-ligand interactor), the receptor for the B cell activating factors BAFF and APRIL (118, 119). The germinal centers, which promote the activation and maturation of the antibody response, are poorly developed in neonates, and production of components of the complement system, is dramatically lower (120). Furthermore, neonatal B cells respond poorly to IL-4, due to a lack of expression of the receptor alpha chain (121). Once they have developed, antigen specific B cell responses decline rapidly, likely because of the lack of B cell survival signals in the bone marrow (120, 122). Infection of neonatal mice recapitulates the weaker antibody response to RSV infection seen in man. Here, IFN-γ produced from NK cells or T cells may act to inhibit the antibody response (123). Therefore, paradoxically too strong a production of certain cytokines could have detrimental effects by reducing the amount of antibody produced in neonates. Importantly, murine studies have found that T follicular helper (Tfh) cell maturation and numbers are lower in neonates, leading to impairment in the T cell help needed to support the B cell response (124). Induction of an appropriate, protective T cell response will be key to the success of a neonatal RSV vaccine.

### Neonatal T cell response to RSV

Development of a T cell response is necessary to provide the help for protective antibody production, and to promote viral clearance, but an unbalanced and dysregulated T cell response to RSV can cause pathology (125). Certainly, those with deficiencies in the T cell response, including primary deficiencies and HIV infection, develop more severe disease following infection (17, 126). In murine models of primary RSV infection, pathology correlates with the peak of the lymphocyte response in the lungs and CD8^+^ lymphocytes are required for viral clearance (127–129). In man, T cells may contribute to the inflammation seen in the lungs in bronchiolitis, however, a paucity of T cells in the lung was reported in one study of children who had died of severe RSV disease (34). The nature, rather than the extent, of T cell responses, and the resultant balance of viral clearance and immunopathology, may be key to determining the outcome of RSV infection.

In primary RSV infection in man and mouse, a type-I response develops, with NK cells, Th1 cells, and CTL all acting as important sources of IFNγ. In the BALB/c murine model, CD8^+^ CTL responses are needed for viral clearance, however, the CD8^+^ response can also lead to enhanced disease (127, 128). A robust systemic and local CD8^+^ T cell response was reported in bronchiolitis, but the response was associated with recovery and not the peak of illness (130, 131). Interestingly, murine studies have revealed that the epitope dominance of the CD8^+^ T cell response differs in RSV infected neonates compared to adults, which may be mediated by profound differences in the DC populations found in the neonatal lung, and suggests the CTL response to infection and vaccination in infants cannot necessarily be predicted from adult studies (72, 132). Furthermore, epitope specific patterns of CTL responses set in infancy could persist into adulthood, with implications for the timing of vaccination.

Although implicated in pathogenic responses to the FI-RSV vaccine, the relevance of Th2 responses in naturally occurring RSV disease is unclear. In infants, an imbalance in the ratio of Th1 and Th2 cytokines in nasal lavage, and a low IFNγ response in BAL and PBMC have been associated with more severe RSV infection (133–135) and IL-4 was particularly elevated in the youngest infants (136). Furthermore, children whose cord blood monocytes were less able to produce IFNγ in response to RSV had an increased frequency of upper respiratory tract infections and respiratory related hospitalizations (137). The presence of elevated concentrations of eosinophil cationic protein has been reported in bronchiolitis, suggesting a polarized type-2 response (136).

There are a number of experimental circumstances under which RSV challenge can, unusually for a viral infection, give rise to Th2-biased lung pathology. Priming with the FI-RSV vaccine or with RSV G-protein expressed in recombinant vaccinia virus, leads to enhanced Th2 driven inflammation upon re-infection (138–140) and infection in IFNγ deficient mice or depletion of NK cells, a primary source of IFNγ, leads to generation of type-2 inflammation to RSV infection (74, 141). Neonatal RSV infection in mice leads to the generation of a type-2 response upon re-challenge in adults (142). Early-life RSV infection may result in a Th2-biased memory and some studies have found a higher proportion of peripheral blood IL-4 secreting RSV-specific T cells following bronchiolitis (143–145), in the absence of an overall bias of PBMC toward Th2 responses (146). However, others find that severe RSV is associated with an exuberant non-eosinophilic, lymphocytic, and neutrophilic response, or by a lack of inflammatory response (31, 33, 34).

Th17 responses have more recently been associated with pathology in RSV. IL-17, the Th17 promoting cytokines IL-1β, IL-6, and IL-23, and CD161^+^ Th17 cells were found to be elevated in the tracheal aspirate of RSV infected infants and IL-17^+^ CD4^+^ cells were elevated in peripheral blood (147–149). In adult mouse models, IL-17, IL-6, and IL-23 are detected in the airways following infection and IL-17 promotes neutrophilic lung inflammation, IL-13 production and a mucogenic response to RSV, and inhibits the CTL response (149–151). Mice deficient in STAT1, which mediates signaling *via* IFNs, develop elevated IL-23, Th17 and mucus responses, suggesting that an early, strong IFN response, which may be lacking in neonates, can reduce the extent of Th17 induction in RSV (151). Furthermore, mice lacking the cytokine IL-27, which promotes Th1 development *via* STAT1 signaling, also developed elevated Th2 and Th17 responses (152). The Th17 response was also elevated in the absence of TLR7, perhaps because of a lack of an IL-12 driven Th1 response (46, 150). IL-17 production by Th cells was also enhanced in the absence of CCR7 (153).

Together this literature suggests the absence of sufficiently strong polarization of T cells toward Th1 may allow the emergence of dysregulated Th17 and Th2 responses to RSV. The neonatal immune system provides such an environment in which Th1 responses are weak and Th2 and Th17 responses are promoted. Neonatal immunization with model antigens tends to give rise to a Th2 memory response (113, 114, 154, 155). As discussed earlier, neonatal DC production of IL-12 in response to TLR ligands, which would promote Th1 responses, is substantially reduced in neonates [reviewed in Ref. (156)]. Interestingly, in neonatal cord blood monocytes, TLR stimulation produced low amounts of IL-12 and IFNα, but substantially enhanced levels of the Th17 promoting IL-1, IL-6, and IL-23 (47, 157) and in peripheral blood monocyte derived DCs, TLR-4, and TLR7 ligands stimulate production of Th17 polarizing cytokines in infant, but not adult DC (148). The production of Th17 promoting cytokines (IL-6 and IL-23) by peripheral blood monocytes peaks at birth and then declines over the first few years of life, whereas, the IL-12 and IFNα production increase with age, with IL-12 not yet reaching adult levels at 2 years (158). In response to RSV itself neonatal cord blood DC in co-culture with autologous T cells produced more IL-4, IL-6, IL-1, and IL-17 and less IL-12p70 and IFNγ than adults, and TGFβ1 was implicated in mediating this difference (159). TGFβ is expressed at high levels in the neonatal lung and it will be important to know how responses of resident lung APC relate to findings in peripheral blood cells. In conclusion, the T cell priming environment in the neonate is likely to favor development of potentially pathogenic T cell responses. Severity, inflammatory context and age at primary RSV infection may all influence this and set the T cell memory to RSV early in life.

Regulatory T cells may be key to providing a balanced T cell response to RSV. In murine models, their depletion leads to enhanced pathology and the generation of Th2 responses (160, 161). In the murine FI-RSV model, inoculation of chemokines into the lung to attract regulatory T cells, was able to reduce severe lung inflammation (101). Circulating levels of regulatory T cells are lower in children with bronchiolitis, although whether this represents a selective recruitment to the airways or a lack of a functional regulatory population is not clear (162). In murine models, lack of IL-10 leads to enhanced pathology in RSV infection (163, 164). IL-10 is found in the airways of bronchiolitic children, is associated with the development of post-bronchiolitic wheeze (PBW), and IL-10 promoter polymorphisms are associated with severe RSV disease in infants (133, 165, 166).

Any future neonatal RSV vaccine will need to use appropriate adjuvants to overcome the neonatal Th2 and Th17 bias and generate appropriate, balanced, protective T cell memory to provide help for the generation of high affinity, high titer antibody in the respiratory tract. Under appropriate conditions, neonates can generate “adult-like” T cell responses, for example at low antigen loads or with a strong Th1 promoting adjuvant (114, 167–169) and recently it has been shown that appropriate use of adjuvants can overcome the impaired Tfh response and boost the generation of the antibody response in neonates (124). Further, although vaccination in early-life results in a poor antibody response there may still be value in vaccinating at this age as a primed adaptive response can subsequently be boosted to generate effective immunity (117). Multiple doses of vaccine using a prime-boost strategy may be a successful approach to inducing immunity to RSV at an early age, although this may be practically and immunologically challenging given the need to achieve protection within the first few months of life.

## Delayed Sequelae of RSV Bronchiolitis

The morbidity burden of RSV infection of infants does not solely encompass the acute disease; for several decades an association has been reported between early-life RSV bronchiolitis and subsequent persistent wheeze (170). This link has been confirmed in several large prospective longitudinal studies (146, 171–175). Sigurs and colleagues have followed 47 children hospitalized for RSV LRTI at under 1 year of age and so far report an association with asthma, recurrent wheeze, and also allergic sensitization persisting to age 18 (175–178). In the Tucson Children’s Respiratory Study, Stein et al. followed children who experienced RSV LRTI before the age of three and found that the RSV cohort were four times more likely to report frequent wheeze than controls by age six (172). In contrast to the Sigurs study, the increased risk attributed to RSV had diminished by age 13 and no association with allergy was found, however in the Tucson cohort the RSV cases were milder LRTI infections with no hospitalization.

There is growing evidence that this association is causal. Wu et al. demonstrated that the risk of asthma in later childhood was related to the age of the child (and thus susceptibility to RSV infection) at the peak of RSV reason (179). Some of the strongest evidence for a causal link comes from a recent well-controlled, randomized, double-blinded trial, in which preventing RSV bronchiolitis by administration of palivizumab to preterm infants was protective against recurrent wheeze in the first year of life (180). Similar protective effects of palivizumab have been observed to three (181) and five (182) years of age, suggesting that an early-life RSV bronchiolitis can indeed have a continuing causal impact beyond infant infection.

Not all infants who experience bronchiolitis will go on to develop asthma or post-bronchiolitic wheeze (PBW) and some infants may be pre-disposed to develop both. Such predisposition may be apparent before RSV infection. In the Copenhagen Prospective Study of Asthma in Childhood (COPSAC) birth cohort of children with family asthma, Chawes et al. reported that the lungs of neonates who went on to develop bronchiolitis already exhibited bronchial hyperresponsiveness prior to any viral infection (183). A recent twin study in Denmark concluded that severe RSV bronchiolitis was a marker for genetic predisposition to asthma (184). Furthermore, a genetic polymorphism in the gene encoding IκBα, a negative regulator of NF-κB, was recently identified to be associated with both RSV and asthma (30). In addition, Gern et al. showed that infants whose cord blood responded with a stronger IFN-γ secretion to RSV stimulation had reduced risk of early PBW, although the infants in this study were all already at increased risk of wheeze with an atopic background (185).

### Potential mechanisms for the delayed sequelae of RSV bronchiolitis

It is not clear how, in those children that are susceptible, RSV bronchiolitis can lead to the development of PBW. A polymorphism in the IL-8 promoter was found at increased prevalence in children who went on to wheeze following bronchiolitis compared to those who did not, suggesting an inherited predisposition to delayed sequelae is linked to differential chemokine expression (186). Ermers and colleagues compared the convalescent blood monocyte cytokine production following infant RSV bronchiolitis and found that high IL-10 and low IL-12 production predicted increased risk of subsequent wheeze (187). However, this prediction extended only to early (up to age 3 years) and not late (to 6 years) PBW, which the study indicated were two separate disease entities.

Because of the difficulty in interpreting clinical studies, investigators have employed animal models to interrogate the effect of an early-life RSV infection on later airway morbidity. One suggested mechanism is that a viral infection in the context of the more Th2-skewed neonatal immune system leads to a permanently dysregulated immune response. In order to probe the interaction between RSV infection and sensitization to allergens, murine RSV infection has been combined with models of allergic asthma. The Th2-biased inflammation and AHR induced by sensitization and challenge with OVA or cockroach allergen was exacerbated when preceded by RSV weeks earlier (188, 189). Whilst these studies were on adult mice, they illustrate an ability of the virus to exacerbate a Th2 response.

We subsequently established that when mice are primed with an RSV infection during a critical neonatal window, there is enhanced “asthma-like” airway inflammation including Th2-biased lymphocyte infiltration and eosinophilia when the mice are re-infected as adults (142). The effect has since been shown to be dependent on TSLP and IL-13 production and cells of both the adaptive and innate immune system (141, 190–194). Manipulation of the immune response during primary infection in the neonate, using the Th1 promoting cytokine IL-18, CpG or IFNγ, reduces pathology following re-infection (141, 195–197). That the outcome of later disease is determined by the age of the mouse on primary infection recapitulates the observation in humans that age of RSV infection and risk of later airway dysfunction are linked (179). That there is a “critical window” in which an environmental insult could permanently alter health status – namely, the developmental origins of health and disease (DoHaD) hypothesis – is an emerging concept relevant to RSV disease (198).

Perhaps the most faithful model of persistent immunological effects is a neonatal infection followed by adult challenge with an unrelated stimulus or allergen. You et al. reported that neonatal RSV alone was enough to cause reduced airway function in adult mice, and when an OVA sensitization/challenge model was additionally employed, the “allergic” phenotype was exacerbated, linked to increased IL-13 levels in the BAL (199). Similarly, using the related pneumovirus of mice (PVM), Siegle found that for development of all the features of childhood asthma, both neonatal PVM infection and allergen sensitization/challenge were needed, dependent on signaling via the IL-4Rα receptor whose ligands include IL-13 (200). Using a slightly different approach, Krishnamoorthy et al. employed a model of maternally transferred tolerance to ovalbumin and observed a breach of tolerance with induction of an asthma-like state when pups were infected with RSV (201). In this model, Tregs were implicated in the pathological state as they themselves acquired a Th2 phenotype and lost the ability to suppress inflammation.

Various other mechanisms have been proposed to explain how RSV might cause long-term airway dysregulation. One controversial theory is that the virus persists or is latent long after the initial inflammation has cleared. Viral RNA has been found in murine lung homogenate up to 100 days after RSV infection (202), and in a guinea pig model persistence of viral RNA correlated with a chronic AHR (203). It is also plausible that continuous viral exposure during a critical window during early-life could modulate immunological development; however, there is no conclusive evidence for this hypothesis.

## Summary

As protective maternal antibody wanes, the infant immune system matures and takes over to counter viral infections and to confer protective immunity. There are still many gaps in our knowledge of infant immunology and of immunity to RSV infection. We do not fully understand why in some infants, protection fails and bronchiolitis develops. The importance of different innate and adaptive immune mechanisms in protection and pathology are poorly understood. It is not clear what constitutes a protective adaptive response to RSV and how to vaccinate to generate it in infants. To produce a high titer, high affinity antibody response appropriate T cell help is needed. However, we still do not fully understand what regulates the differentiation of T cell subsets in infants, and how they interact and establish memory. A central question is why natural B and T cell responses fail to generate protective immunity to RSV even in adults, and whether this is determined by host or viral factors.

Much of our understanding of the immunology of infants has focused on systemic responses or those evident from studies of cord blood. If sIgA is the optimal form of antibody needed to protect against infection, then understanding how postnatal mucosal responses are regulated and develop is an important goal and may aid the rational design of effective vaccines. Further, understanding how environmental factors (such as microbiome, diet, and maternal health) alter the development of the infant immune response and the outcome of RSV infection may ultimately lessen the burden of bronchiolitis. It seems evident that success in this arena would not only benefit infants with bronchiolitis but would also improve long-term respiratory health in later life.

## Conflict of Interest Statement

The authors declare that the research was conducted in the absence of any commercial or financial relationships that could be construed as a potential conflict of interest.
